# Bleitetraethyl und Bleitetramethyl – Addendum: Reevaluierung der BAT‑Werte und Ableitung eines BLW: Beurteilungswerte in biologischem Material

**DOI:** 10.34865/bb7800d10_2ad

**Published:** 2025-06-30

**Authors:** Annette Greiner, Hans Drexler, Andrea Hartwig, MAK Commission

**Affiliations:** 1 Friedrich-Alexander-Universität Erlangen-Nürnberg. Institut und Poliklinik für Arbeits-, Sozial- und Umweltmedizin Henkestraße 9–11 91054 Erlangen Deutschland; 2 Institut für Angewandte Biowissenschaften. Abteilung Lebensmittelchemie und Toxikologie. Karlsruher Institut für Technologie (KIT) Adenauerring 20a, Geb. 50.41 76131 Karlsruhe Deutschland; 3 Ständige Senatskommission zur Prüfung gesundheitsschädlicher Arbeitsstoffe. Deutsche Forschungsgemeinschaft, Kennedyallee 40, 53175 Bonn, Deutschland. Weitere Informationen: Ständige Senatskommission zur Prüfung gesundheitsschädlicher Arbeitsstoffe | DFG

**Keywords:** Bleitetraethyl, Bleitetramethyl, Biologischer Arbeitsstoff-Toleranzwert, BAT-Wert, Biologischer Leitwert, BLW, tetraethyllead, tetramethyllead, biological tolerance value, BAT value, biological guidance value, BLW

## Abstract

The German Senate Commission for the Investigation of Health Hazards of Chemical Compounds in the Work Area (MAK Commission) re-evaluated the data for tetraethyllead [78-00-2] and tetramethyllead [75-74-1] considering all toxicological endpoints and derived a biological guidance value (BLW). Relevant studies were identified from a literature search. In 1994, the previous biological tolerance values (BAT values) for tetraethyllead and tetramethyllead were derived in correlation to the maximum workplace concentration (MAK value) of 50 µg as lead/m^3^. In 2023, the MAK value for organic lead compounds was lowered to 4 µg as lead/m^3^. There are no suitable studies for a corresponding reduction of the BAT value or the derivation of a specific BAT value for tetraethyllead. The former BAT values for tetraethyllead and tetramethyllead in urine are therefore withdrawn.

Tetraethyllead is metabolised to triethyllead, diethyllead and inorganic lead. Tetram﻿eth﻿yllead is metabolised to trimethyllead, dimethyllead and inorganic lead. Exposure to inorganic lead can best be measured via the blood lead concentration. For inorganic lead, a BAT value of 150 µg lead/l blood was derived in 2022. As organic lead compounds are more lipophilic and lead to more pronounced neurological effects than inorganic lead compounds, adverse effects cannot be excluded with certainty by compliance with the value of 150 µg lead/l blood, making a BAT value inapplicable. As a result, for tetraethyllead and tetramethyllead, a BLW of 150 µg lead/l blood is derived. Sampling time is not fixed in the steady state.

**Table:** 

**BAT-Wert (2024)**	**nicht festgelegt**
**BLW (2024)**	**150 µg/l Blut**
	Probenahmezeitpunkt: keine Beschränkung im Fließgleichgewicht
	
**MAK-Wert (2023)**	**0,004 mg/m^3^ (als Blei berechnet)**
Krebserzeugende Wirkung (2024)	Kategorie 4
Fruchtschädigende Wirkung (2024)	Gruppe A
Hautresorption (1966)	H

## Reevaluierung

Für Bleitetraethyl wurde 1994 von der Kommission in Korrelation zu dem damals gültigen MAK-Wert von 50 µg/m^3^ (als Blei berechnet) ein BAT-Wert von 25 µg Diethylblei/l Urin (als Blei berechnet) abgeleitet (Bolt 1996 a). Zusätzlich erfolgte die Angabe eines BAT-Wertes für Gesamtblei im Urin von 50 µg/l Urin. Für Bleitetramethyl wurde ein BAT-Wert von 50 µg Gesamtblei/l Urin festgelegt (Bolt 1996 b). Es wurde in den BAT-Begründungen zudem darauf hingewiesen, dass der BAT-Wert für anorganisches Blei im Blut ebenfalls einzuhalten ist. Im Jahr 2023 wurde der MAK-Wert für organische Bleiverbindungen auf 4 µg/m^3^ (als Blei berechnet) abgesenkt (Hartwig und MAK Commission 2024), so dass auch der BAT-Wert für organische Bleiverbindungen zu reevaluieren ist.

## Neue Studien zur beruflichen Belastung mit organischen Bleiverbindungen

Seit der Veröffentlichung der letzten BAT-Begründungen (Bolt 1996 a, b) sind einige Studien zu organischen Bleiverbindungen publiziert worden.

Kapaki et al. (1998) bestimmten in den Jahren 1991 bis 1994 bei (a) 42 Tankstellenbeschäftigten, (b) 47 Taxifahrern, (c) 47 Busfahrern und (d) 36 altersangepassten Kontrollpersonen (überwiegend Krankenhausmitarbeiter) die Bleikonzentrationen im Blut und führten bei einem Teil der Teilnehmer ein EEG durch. Es ergaben sich keine statistisch signifikanten Unterschiede zwischen den Blutbleikonzentrationen: (a) 56,4 ± 17 µg/l, (b) 59,6 ± 17 µg/l, (c) 58,8 ± 13 µg/l und (d) 57,6 ± 17 µg/l. Zwischen der Kontrollgruppe und potentiell beruflich Exponierten zeigten sich keine Unterschiede bezüglich des Auftretens von abnormen EEG-Befunden und es fanden sich keine statistisch signifikanten Unterschiede im Blutbleispiegel zwischen den Teilnehmern mit normalem oder abnormem EEG. Unterschiede zur Kontrollgruppe ergaben sich bezüglich unspezifischer klinischer Symptome (Hartwig und MAK Commission 2024; Kapaki et al. 1998).

Eingebettet in die obige Studie von Kapaki et al. (1998) wurden 37 Tankstellenbeschäftigte, 41 Bus- sowie 44 Taxifahrer mit Blutbleispiegeln von 55 ± 16 µg/l, 58 ± 13 µg/l bzw. 59 ± 16 µg/l mittels Computertomographie (CT) hinsichtlich kortikaler Atrophie und abnormer Kalzifikationen untersucht. Es ergab sich bei den Tankstellenbeschäftigten ein erhöhtes Odds Ratio (OR) von 6,43 (95-%-Konfidenzintervall (KI): 1,46–28,27) für eine kortikale Atrophie. Abnorme Kalzifikationen traten nicht auf. Die Blutbleispiegel dieser als exponiert betrachteten Gruppen waren im Vergleich zu den 36 nichtexponierten Kontrollpersonen nicht erhöht. CT-Untersuchungen erfolgten in der Kontrollgruppe nicht (Varelas et al. 1999).

Afolabi et al. (1999) untersuchten 35 Personen, die mit Bleitetraethyl Umgang hatten, und 40 Personen, die Tankwagen beluden, im Vergleich zu einer Kontrollgruppe von 36 Grundschullehrern. Die Bleispiegel betrugen 445 ± 104 µg/l Blut, 513 ± 136 µg/l Blut bzw. 346 ± 62 µg/l Blut und lagen damit auch in der Kontrollgruppe weit oberhalb des BAT-Wertes für anorganisches Blei. In den beiden Gruppen mit potentiell beruflich Exponierten fanden sich im Vergleich zur Kontrollgruppe statistisch signifikant häufiger Schlafstörungen, Nervosität und produktiver Husten. Des Weiteren wurden Verschlechterungen in einigen Lungenfunktionsparametern beschrieben.

Yassin und Lubbad (2013) bestimmten bei 72 Tankstellenmitarbeitern den Blutbleiwert und erfragten mittels Fragebogen u. a. das Vorliegen von 19 Symptomen. Der mittlere Blutbleispiegel betrug 114 µg/l. Es wurde mittels t-Test untersucht, ob sich der mittlere Blutbleispiegel der Teilnehmer mit dem jeweiligen Symptom von dem der Teilnehmer ohne das Symptom statistisch signifikant unterscheidet. Dies war der Fall bezüglich Reizbarkeit, Kopfschmerzen, Konzentrationsproblemen, Schlafstörungen und Bluthochdruck. Hingegen konnten bezüglich Müdigkeit, Koma, Krämpfen, Anfällen, Hörverlust, Fallhand/Fallfuß, Libidoverlust, Übelkeit, Dyspepsie, Verstopfung, Bauchschmerzen, Bleisaum am Zahnfleisch, Nierenschmerzen und Unfruchtbarkeit keine signifikanten Unterschiede der mittleren Blutbleispiegel festgestellt werden.

Vural und Duydu (1995) stellten die Bleiausscheidung im Urin von 15 Tetraethylblei-exponierten Tankstellenmitarbeitern differenziert nach Gesamtblei (79,0 ± 41,1 µg Blei/g Kreatinin) und anorganischem Blei (37,3 ± 33,2 µg Blei/g Kreatinin) dar. Eine Kontrollgruppe aus 15 Personen mit einem Wohnort 20 km von Ankara entfernt wies hingegen 5,5 ± 1,6 µg Gesamtblei/g Kreatinin und 3,9 ± 1,6 µg anorganisches Blei/g Kreatinin auf.

In weiteren Publikationen von Duydu et al. (1998) und Duydu und Vural (1998) wurden Gesamtblei im Urin und anorganisches Blei im Urin von 49 Beschäftigten, die in einer Erdölraffinerie Tanks befüllten, sowie von 50 Motormechanikern, die Kraftstoff zum Reinigen ihrer Hände benutzten, und von 42 Beschäftigten im Service von Tankstellen untersucht. Als Kontrollgruppe dienten 35 nicht beruflich exponierte Bewohner von Ankara. Es ergaben sich für die Beschäftigten der Erdölraffinerie 91,1 ± 37,1 µg Gesamtblei/g Kreatinin und 65,8 ± 24,6 µg anorganisches Blei/g Kreatinin, für die Motormechaniker 67,3 ± 42,8 bzw. 48,4 ± 29,3 µg Blei/g Kreatinin, für die Beschäftigten der Tankstellen 74,2 ± 41,1 bzw. 53,9 ± 35,5 µg Blei/g Kreatinin und für die Kontrollgruppe 50,5 ± 17,4 bzw. 45,9 ± 14,1 µg Blei/g Kreatinin.

Duydu und Vural (1998) publizierten von 49 Erdölraffinerie-Mitarbeitern, 50 Motormechanikern und 35 männlichen Kontrollpersonen zusätzlich Messungen der Aminolävulinsäure (ALA) im Urin. Diese zeigte in den exponierten Gruppen kreatininadjustiert einen statistisch signifikanten Anstieg.

Schwartz et al. (1993) untersuchten 222 Beschäftigte eines Bleitetraethyl-produzierenden Betriebs mittels einer neuropsychologischen Testbatterie, Tests der Riechfähigkeit und der peripheren Vibrationsschwelle sowie eines Fragebogens zu psychiatrischen Symptomen. In der Luft von 29 Expositionsbereichen wurden Konzentrationen von 4–119 µg/m^3^ für organisches Blei und 1–56 µg/m^3^ für anorganisches Blei geschätzt. Anhand der kumulativen Bleiexposition und der Expositionsdauer wurden die Beschäftigten in Expositionsgruppen eingeteilt. Es fanden sich Assoziationen zwischen schlechteren neuropsychologischen Testergebnissen und höherer kumulativer Bleiexposition und Expositionsdauer. Der Vergleich mit 62 nichtexponierten Beschäftigten der gleichen Einrichtung ergab einen Zusammenhang zwischen dem Blutbleispiegel und der Reaktionszeit (simple visual reaction time). In der Gruppe der Exponierten betrug der Bleispiegel im Blut 205 ± 100 µg/l bei einer Spannweite von 10 bis 510 µg/l. Die Beschäftigten wiesen eine mittlere maximale Urinkonzentration von 143 ± 130 µg Blei/l (Bereich 0–1035 µg/l) auf. Für die Vergleichsgruppe lagen keine Blut- oder Urinmessungen vor. Die Daten deuten auf eine nichtlineare Beziehung hin: Ab einem Blutbleispiegel von mehr als 300 µg/l zeigten die Probanden laut den Autoren einen erheblichen Anstieg der durchschnittlichen Reaktionszeit (Balbus et al. 1997). Eine weitere Auswertung des Kollektivs der 222 Beschäftigten ergab positive Zusammenhänge von Alter und Zigarettenrauchen mit den Blutbleispiegeln. Unerwartet war hingegen, dass Alkoholkonsum mit niedrigeren Blutbleispiegeln korrelierte. Arbeiter, die Alkohol konsumierten, zeigten bei zunehmender Exposition gegen organisches Blei einen geringeren Anstieg der Blutbleispiegel als Arbeiter, die keinen Alkohol tranken. Eine solche Effektmodifikation ließ sich mit anorganischen Bleiverbindungen nicht beobachten (McGrail et al. 1995). In einer Fallserie von Mitchell et al. (1996) wurden 58 Beschäftigte desselben Betriebes wegen ihrer Besorgnis aufgrund ihrer Bleiexposition auf eigenen Wunsch in einer universitären arbeitsmedizinischen Einrichtung untersucht. Der Arbeitgeber stellte für 48 Personen Angaben zu früheren Blut- und Urinbleispiegeln bereit, wobei der mittlere Blutbleispiegel bei 261 ± 91 µg/﻿l, die mittlere Urinbleikonzentration bei 51,2 ± 18,8 µg/l und die maximalen Urinbleikonzentrationen zwischen 36 µg/﻿l und 500 µg/l lagen. Die aktuellen Blutbleispiegel betrugen im Mittel 194 ± 65 µg/l (n = 39). Die am häufigsten genannten Symptome waren Gedächtnisverlust, Gelenkschmerzen, Schlafstörungen, Reizbarkeit, Parästhesien, Müdigkeit und Albträume. Bei einem Teil der Patienten wurde aufgrund von Symptomen im Bereich des peripheren Nervensystems oder auffälligen Befunden bei der körperlichen Untersuchung zusätzlich die Nervenleitfähigkeit untersucht. Bei elf von ihnen wurde eine Polyneuropathie gesichert. Zumindest eine Auffälligkeit wurde bei 22 der 31 untersuchten Patienten gefunden (siehe Hartwig und MAK Commission 2024).

Mehrere Publikationen befassen sich mit Untersuchungen an Personen, die in der Vergangenheit einer Mischexposition gegen organisches und anorganisches Blei ausgesetzt waren:

Mehrere Studien wurden an Beschäftigten durchgeführt, die gegen organisches Blei exponiert waren. In einer ersten Studie wurden 543 mit organischen Bleiverbindungen Tätige mittels neuropsychologischer Testung untersucht. Die letzte berufliche Exposition lag im Durchschnitt 17,8 Jahre zurück. Zur Abschätzung der früheren Bleibelastung wurde die aktuelle Bleikonzentration in der Tibia herangezogen. Unter der Annahme einer Halbwertszeit von 27 Jahren und einer Elimination erster Ordnung wurde die Tibia-Bleikonzentration im letzten Beschäftigungsjahr berechnet. Der ermittelte Durchschnittswert lag bei 23,7 ± 17,4 µg Blei/g Knochenmineral. Die abgeschätzte Bleikonzentration der Tibia im letzten Beschäftigungsjahr erwies sich als signifikant negativ assoziiert mit der Leistung in mehreren neuropsychologischen Tests: Wechsler Adult Intelligence Scale – Revised vocabulary subtest (Wechsler-Intelligenzskala für Erwachsene – überarbeiteter Untertest zum Wortschatz), serial digit learning test (serieller Ziffernlerntest), Rey Auditory-Verbal Learning Test (immediate recall and recognition) (Rey auditiv-verbaler Lerntest (sofortiges Erinnern und Erkennen)), Trail Making Test B (Zahlen-Verbindungs-Test (Version B)), finger tapping (Finger-Tapping), Purdue pegboard (Purdue Stecktafel Test: 30 Sekunden; rechte, linke, beide Hände; Montage von Stift und Unterlegscheibe) und Stroop-Test (Farb-Wort-Interferenz-Test) (Stewart et al. 1999).

Schwartz et al. (2000) untersuchten in diesem Kollektiv bei 535, wie oben beschrieben, ehemals Exponierten sowie bei 118 Kontrollpersonen den zeitlichen Verlauf der kognitiven Leistungen über vier Jahre. Als Expositionsmaß wurde der aktuelle Blutbleispiegel im ersten Untersuchungsjahr gemessen und mit der abgeschätzten Tibia-Bleikonzentration in Beziehung gesetzt. Bei den Teilnehmern, die durchschnittlich seit 16 Jahren nicht mehr beruflich exponiert waren, lag der aktuelle Blutbleispiegel bei 46 ± 26 µg/l. Verglichen mit einer Kontrollgruppe (n = 118) schnitten die bleibelasteten Beschäftigten in drei Tests (visuell-konstruktive Fähigkeit, verbales Gedächtnis und Lernen) über die Jahre schlechter ab. Eine hohe Tibia-Bleibelastung korrelierte mit einer Abnahme der kognitiven Fähigkeiten in sechs Tests (verbales Gedächtnis und Lernen, visuelles Gedächtnis, Ausführungsfähigkeit und manuelle Geschicklichkeit) (siehe Hartwig und MAK Commission 2024).

In einer weiteren Studie untersuchten Schwartz et al. (2007) an 532 ehemals Exponierten die Beziehungen zwischen kognitiven Funktionen und Hirnvolumina, die mittels Magnetresonanztomographie bestimmt wurden. Die Studie fand statistisch signifikante Zusammenhänge zwischen der Bleibelastung und Veränderungen in spezifischen Hirnregionen.

Eine weitere longitudinale Untersuchung im Jahr 2010 an Teilnehmern aus demselben Kollektiv ergab keine Assoziationen zwischen den Tibia-Bleikonzentrationen und Veränderungen der Hirnvolumina oder Läsionen in der weißen Substanz über einen durchschnittlichen Untersuchungszeitraum von fünf Jahren (Schwartz et al. 2010).

Weitere Informationen u. a. auch zu den Publikationen von Stewart et al. (2002, 2006) sowie eine Zusammenfassung der Studie von Stewart und Schwartz (2007) finden sich bei Hartwig und MAK Commission (2024).

In einigen Kleinflugzeugen wird verbleites Flugbenzin verwendet. In zwei Studien wurden höhere Blutbleispiegel bei Kindern beobachtet, die in der Nähe von Flughäfen in North Carolina (Miranda et al. 2011) und Kalifornien (Zahran et al. 2022) leben. Zahran et al. (2022) untersuchten den Zusammenhang zwischen der Luftbleikonzentration am Flughafen und der kindlichen Blutbleikonzentration. Die Modellberechnung ergab, dass ein Anstieg der Luftbleikonzentration um 1 μg/m^3^ die Blutbleispiegel von Kindern um 40,5 μg/l erhöht. Dieser Zusammenhang basiert auf einer zweimonatigen Messung der Luftbleibelastung vor der Blutentnahme. Beschränkt man die Messung der Luftbleibelastung auf die letzten 30 Tage vor der Blutentnahme, verringert sich die geschätzte Zunahme auf 24,5 μg/l (95-%-KI: 9,3–39,6).

## Reevaluierung des BAT-Wertes

Seit der Veröffentlichung der letzten Begründung wurden keine Studien publiziert, die ausreichend belastbare Ergebnisse liefern, um einen Beurteilungswert für Bleitetraethyl oder Bleitetramethyl abzuleiten.

So wurden in einigen der dargestellten Studien keine ausreichend validen Biomonitoring-Daten berichtet, Angaben zu Effekten oder der Luftkonzentration waren oftmals nicht vorhanden. In einigen Studien lagen die Bleikonzentrationen oberhalb des bisherigen BAT-Wertes.

In den Untersuchungen von Kapaki et al. (1998) und Varelas et al. (1999) sind die Blutbleispiegel in den exponierten Gruppen im Vergleich zur Kontrollgruppe zum Untersuchungszeitpunkt nicht erhöht. Es fehlen Angaben zur früheren Bleiexposition. Andere Substanzen wie weitere Kraftstoff-Inhaltsstoffe können als Ursache der beobachteten Befunde nicht ausgeschlossen werden. Diese Publikationen liefern daher keine Erkenntnisse, die für die Ableitung eines Grenzwertes für Blei in biologischem Material relevant sind.

Auch bei der Arbeit von Yassin und Lubbard (2013) ist zu bedenken, dass Ottokraftstoff unter anderem diverse Kohlenwasserstoffe enthält. Kraftstoff-Inhaltsstoffe wie Benzol, Toluol, Xylol und *n-*Hexan (vgl. ATSDR 1995) sind als Ursache von berichteten Symptomen zu erwägen. Auch das Alter als Einflussfaktor ist sowohl hinsichtlich des Bleiwertes als auch der Häufigkeit einiger Symptome zu bedenken. Eine Kontrollgruppe wurde nicht in die Studie einbezogen. Inwiefern sich die statistische Analyse mit multiplen t-Tests (ohne Korrektur für multiples Testen) auf die Signifikanz der Ergebnisse auswirkt, kann ebenfalls nicht endgültig beurteilt werden. Gesundheitliche Effekte von organischen Bleiverbindungen bei Blutbleiwerten unterhalb von 150 µg/l können deshalb nicht mit hinreichender Sicherheit als belegt gelten. In der Zusammenschau lässt sich anhand dieser Studie kein Beurteilungswert für Bleitetraethyl oder Bleitetramethyl ableiten.

In vielen der zitierten Studien wurden Kollektive mit einer Mischexposition gegen organisches und anorganisches Blei untersucht (z. B. in Studien von Stewart et al. (1999), Schwartz et al. (1993) und weiteren Untersuchungen an diesem Kollektiv). Dies gilt auch für Kollektive, deren Bleibelastung vorrangig aus der Verwendung verbleiten Benzins als Kraftstoff stammte. Wie bei Hartwig und MAK Commission (2024) beschrieben, wird ein Großteil des Bleitetraethyls und Bleitetramethyls im verbleiten Benzin während des Verbrennungsvorgangs zu anorganischem Blei umgesetzt. Dieser Aspekt muss z. B. bei Taxifahrern und Busfahrern, aber auch bei den Untersuchungen an Kindern mit Wohnort im Umfeld von Flughäfen berücksichtigt werden. Aus den Studien mit Mischexposition lässt sich kein BAT-Wert für Bleitetraethyl oder Bleitetramethyl ableiten.

Auch die Studien an ehemals Bleiexponierten können für die Ableitung eines BAT-Wertes nicht herangezogen werden, da u. a. Biomonitoring-Ergebnisse in Blut oder Urin aus dem Zeitraum der Exposition nicht vorliegen und es sich auch hier um eine Mischexposition aus organischem und anorganischem Blei handelt.

Neue Studien zu organischen Bleiverbindungen, die die Ableitung eines spezifischen BAT-Wertes für Bleitetraethyl oder Bleitetramethyl ohne Korrelation zum MAK-Wert ermöglichen würden, liegen somit nicht vor.

Da im Jahr 2023 der MAK-Wert für organische Bleiverbindungen von 50 auf 4 µg/m^3^ (als Blei) abgesenkt wurde, der bisherige BAT-Wert für Bleitetraethyl in Korrelation zum früher gültigen MAK-Wert von 50 µg/m^3^ abgeleitet wurde und keine geeigneten Studien für eine entsprechende Absenkung eines BAT-Wertes oder die Ableitung eines spezifischen BAT-Wertes für Bleitetraethyl vorliegen,

**wird der BAT-Wert für Bleitetraethyl ausgesetzt.**

Bereits in der vorangegangenen Begründung zu Bleitetramethyl wurde auf die umfangreichen Analogien zwischen Bleitetraethyl und Bleitetramethyl verwiesen und der für Bleitetraethyl evaluierte BAT-Wert für eine Gesamtbleiausscheidung im Urin auch auf Bleitetraethyl/Bleitetramethyl-Mischexpositionen bezogen.

**Daher wird auch der BAT-Wert für Bleitetramethyl ausgesetzt.**

## Evaluierung eines Biologischen Leitwertes (BLW) im Blut

Bleitetraethyl wird im Körper dealkyliert. Im Urin finden sich Triethylblei, Diethylblei und anorganisches Blei (Ya﻿ma﻿mu﻿ra et al. 1981). Auch Bleitetramethyl erfährt eine Dealkylierung zu Trimethylblei, Dimethylblei und anorganischem Blei (Greim 1995). Der beste Parameter zur Abbildung einer Belastung mit elementarem und anorganischem Blei ist die Blutbleikonzentration. Auch der MAK-Wert für anorganische Bleiverbindungen wurde daher auf der Basis des BAT-Wertes für anorganisches Blei im Blut abgeleitet (Hartwig und MAK Commission 2022).

Bereits in der BAT-Begründung zu Bleitetraethyl von 1996 wurde zusätzlich auf die Notwendigkeit der Einhaltung des BAT-Wertes für anorganisches Blei im Blut hingewiesen (Bolt 1996 a). Diesbezüglich hat die Reevaluierung keine Änderung ergeben.

Organische Bleiverbindungen wie Bleitetraethyl und Bleitetramethyl zeigen aufgrund ihrer Lipophilie stärkere neurotoxische Effekte als Blei-Ionen (Hartwig und MAK Commission 2024). Daher kann es nicht als hinreichend gesichert gelten, dass bei Einhaltung des für elementares und anorganisches Blei abgeleiteten BAT-Wertes im Blut bei Exposition gegen Bleitetraethyl oder Bleitetramethyl keine adversen Effekte auftreten. Entsprechende epidemiologische Studien für organische Bleiverbindungen fehlen, so dass der Wert von 150 µg Blei/l Blut bei Exposition gegen Bleitetraethyl oder Bleitetramethyl nur als BLW aufgestellt werden kann.

Es wird daher für Bleitetraethyl und Bleitetramethyl ein

**BLW von 150 µg Blei/l Blut abgeleitet.**

Aufgrund der langen biologischen Halbwertszeit von Blei im Menschen ist der Probenahmezeitpunkt nicht an einen definierten Zeitpunkt gebunden („keine Beschränkung im Fließgleichgewicht“).
